# Traumatic Dental Injuries among Adolescents and Young Adults in Iași, Romania: Legal and Medical Perspectives

**DOI:** 10.3390/dj12090282

**Published:** 2024-09-02

**Authors:** Giuvara Constantin Răzvan, Victor Vlad Costan, Otilia Boisteanu, Adina Armencia, Mihai Ciofu, Carina Balcos, Gabriela Calin, Diana Bulgaru Iliescu, Loredana Liliana Hurjui

**Affiliations:** 1 Department of Medical Disciplines, Faculty of Dentistry, “Grigore T. Popa” University of Medicine and Pharmacy, 700115 Iasi, Romania; dr.razvan.giuvara@gmail.com (G.C.R.); victor.costan@umfiasi.ro (V.V.C.); otilia.boisteanu@yahoo.com (O.B.); adina.armencia@umfiasi.ro (A.A.); ciofu.miha@yahoo.com (M.C.); 2 Faculty of Dentistry, Apollonia University, 700115 Iasi, Romania; 3 Department of Medical Disciplines, Faculty of Medicine and Pharmacy, “Grigore T. Popa” University of Medicine and Pharmacy, 700115 Iasi, Romania; bulgarudiana@yahoo.com; 4 Department of Morpho-Functional Sciences I, Discipline of Histology, “Grigore T. Popa” University of Medicine and Pharmacy, University Street 16, 700115 Iasi, Romania; loredana.hurjui@umfiasi.ro

**Keywords:** dental trauma, periodontal trauma, adolescents, young adults

## Abstract

Adolescents and young adults’ active lifestyles make dental trauma a significant medical concern. Aim: This study aims to assess the etiology, frequency, and localization of dental and periodontal trauma in adolescents and young adults, along with these individuals’ predisposition based on age, gender, and residence. Materials and Methods This retrospective study included 109 adolescents and young adults from a database of the Emergency and Oral and Maxillofacial Surgery department at “Sf. Spiridon” Hospital, Iasi, Romania. The collected data included demographic details, mechanisms of dental and periodontal trauma, treatment approaches, and clinical outcomes. Statistical analysis was performed using SPSS 26.0, with comparisons based on age, gender, and residence. Results: Enamel fractures (23.9%) and enamel-dentin fractures without pulp exposure (20.2%) were the most frequent dental injuries. Concussion (58.7%), subluxation (21.1%), luxation, avulsion (7.3%), and extrusion (5.5%) were common periodontal injuries. The anterior region of the dental arch accounted for the majority (60.6%) of injuries. Adolescents under 17 years were more prone to enamel-dentin fractures without pulp exposure (23.8%), enamel-dentin fractures with pulp exposure (20.6%), and root fractures (20.6%), while young adults over 18 years had a higher incidence of enamel fractures (32.6%) and crown-root fractures (30.4%). Falls (66.7%) were the predominant cause of trauma for female patients, whereas male patients were more often injured during sports activities (49.3%). The younger age group was 0.29 times more likely to sustain enamel-dentin fractures without pulp exposure (*p* = 0.049, OR = 0.291) and root fractures (*p* = 0.047, OR = 0.241). Conclusions: Traumatic dental injuries are more prevalent in young adults compared to adolescents, with falls and sports activities being the most common causes, particularly among males. These findings emphasize the need for targeted preventive programs aimed at reducing the incidence of dento-periodontal trauma in these age groups.

## 1. Introduction

Dental trauma represents a significant and common occurrence among adolescents and young adults, largely due to their active and often risk-taking lifestyles. While these injuries may not always be severe, they can lead to complex medico-legal issues that require careful consideration. In forensic settings, traumatic dental injuries are the most frequently observed type of dental injury, highlighting their prevalence and importance in both clinical and legal contexts. The likelihood of experiencing dental trauma varies with several factors, including the victim’s age, gender, ethnicity, and socioeconomic background. Typically, the incidence of traumatic dental injuries first becomes prominent around the age of one, coinciding with the onset of walking and the development of motor skills. This incidence then reaches a peak during the school years, a period characterized by increased physical activity and participation in sports [1,2].

The rising prevalence of these injuries is also closely linked to an increase in participation in contact sports among adolescents and young adults. As more individuals engage in activities such as football, basketball, hockey, and martial arts, the risk of dental trauma increases proportionally. This trend underscores the importance of protective measures such as mouthguards in preventing dental injuries during sports activities. 

The prevalence of dental traumas varies widely across different populations and age groups. Studies conducted thus far have shown a prevalence ranging from 2% to 59%, with the age range of 17–25 years showing a prevalence between 17 and 20%. This wide range in reported prevalence can be attributed to differences in study methodologies, population characteristics, and definitions of dental trauma used in various research studies [3,4,5,6,7].

Dental trauma can arise under various scenarios, most commonly through mechanical forces, but also through physical and chemical means. Often, these injuries result from a direct impact on the teeth or jaw, particularly the chin, with the force then transmitting to the opposing teeth. Alternatively, a fall that impacts the face against an uneven surface can cause dental injuries. Such dental injuries can occur from a direct mechanism or an indirect mechanism, where forced closure of the jaw results in an interaction between the upper and lower dental arches. This indirect action can lead to coronal fractures in the back of the mouth, as well as root and condyle fractures, and damage to the jaw’s symphysis [3,4,5,6].

The study of dental trauma in adolescents and young adults is of particular importance due to the high prevalence in this age group and the potential for long-term consequences. By understanding the patterns, causes, and risk factors associated with dental trauma in this population, healthcare providers can develop more effective prevention strategies and treatment protocols. Furthermore, this knowledge can inform public health policies aimed at reducing the incidence of dental trauma and improving oral health outcomes in young people [8,9].

The impact of dental trauma may extend beyond the teeth to the hard structure, potentially involving the pulp cavity or periodontal components. Frequently, these injuries are compounded by damage to the orofacial soft tissues or jawbones, exacerbating the severity of the trauma and increasing the risk of fatal outcomes. Therefore, this study aimed to evaluate the etiology, frequency, and location of dental and periodontal trauma, as well as the predisposition of adolescents and young adults to these types of traumas according to age, gender, and residence.

## 2. Materials and Methods

### 2.1. Type of Study and Data Collection

This retrospective study included 109 adolescents and young adults selected from a database of the Emergency and Oral and Maxillofacial Surgery department at the “Sf—Spiridon” Hospital in Iasi between March 2022 and December 2023. Specialists at the Oral and Maxillofacial Surgery Unit performed data recording. This study was carried out after it was approved by the Ethics Commission of UMPh Grigore T. Popa Iasi (no. 9532/15.06.2020). Included in the collected data were detailed demographic information (age, gender, and environment of residence), mechanisms of dento-periodontal trauma (domestic accidents, motor accidents, and animal-caused injuries), wound management techniques, therapeutic approaches, and dental patient outcomes. These documents were acquired to guarantee a thorough comprehension of every case [10].

The study design allowed for comparisons of the collected data based on age, gender, and residence, enabling the identification of potential risk factors and patterns associated with dental and periodontal trauma in this population.

### 2.2. Selection Criteria of the Cases

This study included patients with maxillofacial trauma associated with dental trauma (enamel fracture, enamel-dentin fracture without pulp exposure, enamel-dentin fracture with pulp exposure, corona-radicular fracture, root fracture, and alveolar fracture) and patients with periodontal trauma. The selected age group was 10–25 years, considering that this age group has an increased risk of dental trauma due to sports activities and accidents specific to this period of life [11,12].

### 2.3. Evaluation of Medico-Legal Aspects 

Forensic etiological aspects such as the manner and outcome of injuries were assessed in detail. Dental traumas were classified according to international guidelines, following the classification proposed by Andreasen and the ICD-DA classification of the World Health Organization (1992). This uniform classification was applied to ensure consistency in data reporting and analysis [13].

Medico-legal etiological aspects like pattern, manner, and outcome of injuries were evaluated. In this research, the etiology of dental trauma was classified into four distinct categories: falls, sports accidents, domestic accidents, and motor vehicle accidents. Falls are trauma resulting from accidental slips or falls not involving sports or domestic activities. Sports accidents were those that occurred during participation in organized or recreational sports activities. Domestic accidents included all injuries that occurred within the home, and motor vehicle accidents were defined as injuries resulting from vehicular collisions. Dental trauma was classified as enamel fracture, enamel-dentin fracture without pulp exposure, enamel-dentin fracture with pulp exposure, crown-root fracture, root fracture, and periodontal trauma such as concussion, subluxation, luxation, extrusion, avulsion, alveolar fracture. Legal participation was strongly associated with the kind of treatment given. Despite being less invasive and cost-effective, conservative therapies resulted in moderate legal compensation. However, dental extractions and implants, which are complex and costly treatments, were associated with substantial legal compensation. Conservative therapies included immobilizing teeth affected by dislocation or subluxation with flexible splints to allow periodontal healing, performing endodontic treatment on teeth with coronal fractures that expose the pulp to prevent infection and preserve dental function, and direct restorations using composites to repair minor fractures of the enamel and dentin.

### 2.4. Clinical and Paraclinical Evaluation 

For the evaluation of multi-traumatized patients, computed tomography (CT) or orthopantomography (OPT) was used, as these are essential for the precise identification of the fracture pattern. These imaging methods are well documented in the specialized literature as the gold standard for the evaluation of dental and maxillary trauma [14].

### 2.5. Statistical Analysis

Data analysis was conducted using SPSS software version 26 (SPSS, Inc., Chicago, IL, USA), a powerful tool for complex statistical computations. The results were expressed as frequencies and mean with standard deviations (SD), providing a clear overview of the data distribution. The Chi-square test was employed to compare proportions between different etiological causes of dento-periodontal injuries according to age, gender, and residence. This test is particularly useful for categorical data, allowing for the identification of significant associations between variables.

To evaluate the degree of exposure to various risk factors, multivariate regression analysis was performed. This advanced statistical technique enables the simultaneous examination of multiple variables, providing insights into the complex interplay of factors contributing to dental and periodontal trauma. A *p*-value of less than 0.05 was considered statistically significant for all analyses. This threshold is widely accepted in medical research and ensures that the observed relationships are unlikely to have occurred by chance.

## 3. Results

Only 109 cases were selected after applying the selection criteria. A total of 57.8% of the participants were 14–17 years, and 42.2% were 18–25 years. More than half of the subjects were male (61.5%), and 54.1% of the subjects came from urban areas (Table 1).

**Figure 1:**
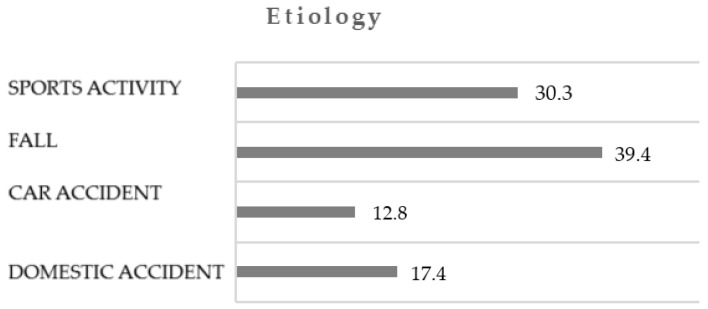
Distribution of cases according to trauma etiology.

The distribution of cases according to the etiology of dental traumas was as follows: 39.4% were caused by falls, 30.3% by sports activities, 17.4% by domestic accidents, and 12.8% by car accidents (Figure 1).

**Figure 2:**
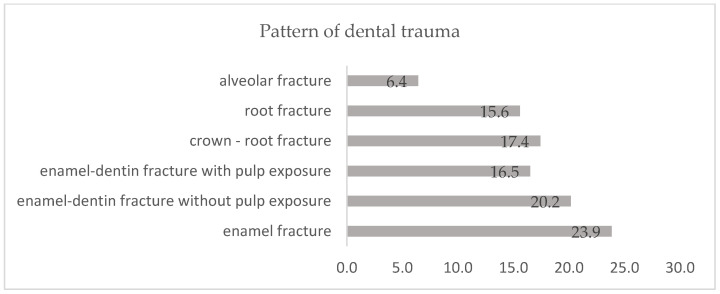
Distribution of dental trauma cases.

Figure 2 shows the types of dental trauma detected: 23.9% of the cases were enamel fractures, 20.2% were enamel-dentin fractures without pulp exposure, 17.4% were crown-radicular fractures, 16.5% were enamel-dentin fractures with pulp exposure, and 6.4% were alveolar fractures.

**Figure 3:**
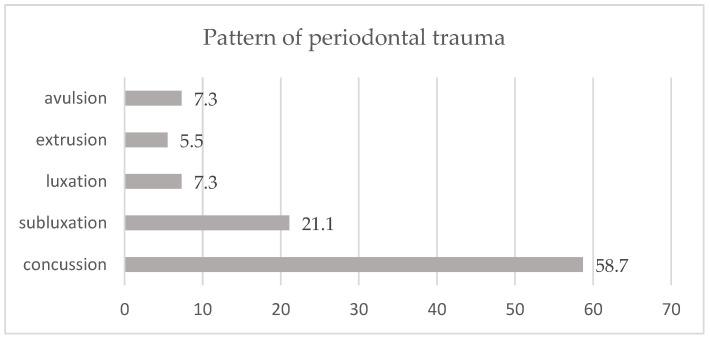
Distribution of periodontal trauma cases.

**Figure 4:**
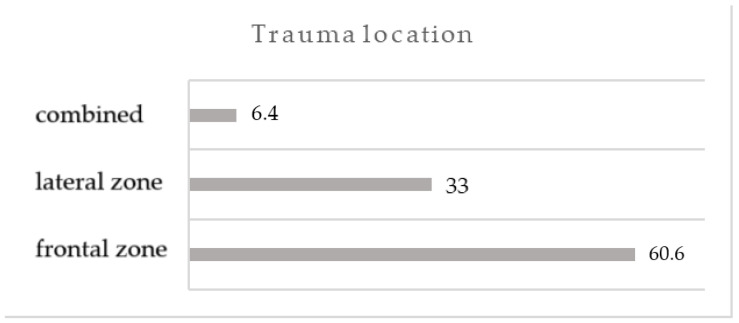
Distribution of trauma cases according to location.

The distribution of cases according to the type of periodontal trauma was as follows: concussion (58.7%), subluxation (21.1%), luxation and avulsion (7.3%), and extrusion (5.5%) (Figure 3). As for the location of the traumatic injuries, we can say that 60.6% of the injuries were in the frontal area of the dental arch (Figure 4).

Table 2 shows the results of the analysis regarding the distribution of cases according to etiology, pattern of dental trauma, pattern of periodontal trauma, trauma location, and type of treatment vs. age, gender, and residence. Depending on the age, the statistical analysis indicates that the participants suffered from traumas caused by falls and sports activities. Those under the age of 17 had more cases of enamel-dentin fracture without pulp exposure (23.8%), enamel-dentin fracture with pulp exposure (20.6%), and root fracture (20.6%), while the participants aged over 18 years had more cases of enamel fracture (32.6%) and crown-root fracture (30.4%). More than half of the periodontal lesions were contusions for both age categories (*p* = 0.004), which were located more in the frontal area (63.5%, 56.5%, *p* = 0.019), and the treatments that were carried out were conservative (Table 2).

Female subjects suffered from dental and periodontal trauma caused by falls (66.7%), while male subjects had trauma caused by sports activities (49.3%). Female subjects had more dental traumas, such as enamel fracture (23.8%) and enamel-dentin fracture without pulp exposure (31%), contusions (59.5%), and subluxations (28.6%), and these injuries were located more in the frontal area. Male subjects had more cases of enamel fracture (23.9%) and enamel-dentin fracture with pulp exposure (22.4%), contusions (58.2%), and avulsions (10.4%), which were located more in the frontal area of the arch 62.7%) (Table 2). These gender-specific patterns highlight the need for targeted prevention strategies that address the unique risk factors and activity profiles of male and female adolescents and young adults. For example, fall prevention programs may be particularly beneficial for females, while sports-related injury prevention might be more critical for males.

The multivariate regression analysis provided valuable insights into the risk factors associated with specific types of dental trauma. 

The results showed that younger participants were 0.29 times more likely to experience enamel-dentin fracture without pulp exposure (*p* = 0.049, OR = 0.291) and 0.241 times more likely to suffer from radicular fractures (*p* = 0.047, OR = 0.241). This increased risk among younger individuals may be attributed to factors such as ongoing dental development, increased participation in high-risk activities, or less developed protective reflexes (Table 3).

Additionally, the analysis revealed that subjects from urban areas were 0.241 times more prone to radicular fractures compared to their rural counterparts (*p* = 0.043, OR = 0.235). This urban–rural disparity could be related to differences in lifestyle, access to recreational facilities, or exposure to certain environmental hazards. Table 3 presents the detailed results of this multivariate regression analysis, offering a comprehensive view of the predisposition to dental trauma according to age, gender, and residence.

## 4. Discussion

As dental professionals and healthcare policymakers, we find ourselves at the forefront of a challenge that not only impacts individual well-being but also carries significant socioeconomic implications. The landscape of oral health in our youth is changing rapidly and is influenced by a complex interplay of factors that we are only beginning to fully understand.

Recent global trends paint a concerning picture. Age-specific prevalence rates are climbing, with studies showing a 15% increase in dental trauma cases among 15–25-year-olds over the past decade. The emergence of new risk factors, such as the popularity of extreme sports and the unexpected effects of social media challenges, suggests that the long-term effects of early dental trauma are more serious than previously believed and may have an impact on general health and quality of life well into adulthood [14]. Long-term consequences of early dental trauma are more severe than previously thought, potentially affecting overall health and quality of life well into adulthood [15,16,17,18]. 

Recovery from the effects of traumatic dental injuries is a complex matter because not all dental trauma methods are supported by adequate research, and pain management, tetanus shots, and antibiotics are examples of supplementary care that must be taken into account [19].

The implementation of public health policy is of major importance in the field of dental trauma because it addresses preventive measures and diminishes the effects of traumatic brain injuries. These dental traumas will be prevented by programs, research, and development to promote a better healthcare system [20]. 

Dental trauma, also known as traumatic dental injury, refers to damage inflicted on the teeth and the associated hard and soft tissues in and around the mouth and oral cavity. This type of injury is typically abrupt, occurring due to unforeseen, accidental circumstances and often necessitates immediate emergency care. It is not classified as a disease but rather an injury because of various inevitable risk factors in everyday life. While certain groups may be more prone to these injuries (children, adolescents, and young adults), no one is completely exempt from risk during their daily activities. Such injuries can significantly alter the life of a person who has previously only needed routine dental check-ups and cleaning and can lead to extensive dental treatments [17,18,19,20,21]. 

In our study, the causes of dental trauma were as follows: falls accounted for 39.4%, sports activities 30.3%, domestic accidents 17.4%, and car accidents 12.8%. Popular national sports like rugby, judo, and football, which involve intense physical contact including grappling and tackling, were significant contributors to sports-related injuries. Additionally, other high-impact activities and accidents, such as fights, assaults, and bicycle and motor vehicle accidents, have been commonly reported in various studies. Our literature review indicated that the home is the most frequent location for such injuries [17,18,19]. Our results are consistent with those reported in the specialized literature [22,23,24,25,26].

Uncomplicated crown fractures are the most common type of dental injury in permanent teeth, which raises concerns for epidemiological studies. Often, these injuries are not considered severe enough to require treatment, which might result in an underestimation of the actual prevalence of dental trauma. According to our research, the distribution of dental trauma types was as follows: 23.9% were enamel fractures, 20.2% were enamel-dentin fractures without pulp exposure, 17.4% were crown-radicular fractures, 16.5% were enamel-dentin fractures with pulp exposure, and 6.4% involved alveolar fractures.

The age-related analysis in our study revealed distinct patterns in the types of dental injuries sustained. Among individuals under 17 years old, the predominant injuries were enamel-dentin fractures without pulp exposure (23.8%), enamel-dentin fractures with pulp exposure (20.6%), and root fractures (20.6%). By contrast, participants over 18 years old more commonly experienced enamel fractures (32.6%) and crown-root fractures (30.4%). These variations between age groups emphasize the importance of implementing age-specific preventive strategies and treatment approaches to address the different risks and types of dental trauma.

The distribution of types of dental trauma varies significantly by age and gender, with trauma more common among young men and caused predominantly by sports activities. These correlations emphasize the need for preventive measures specific to the demographic groups identified as the most vulnerable.

In our study, the distribution of periodontal trauma types was as follows: concussions accounted for 58.7% of cases, subluxations for 21.1%, luxations and avulsions for 7.3%, and extrusions for 5.5% (as shown in Figure 3). Regarding the location of the injuries, 60.6% occurred in the frontal area of the dental arch. The prevalence of luxation injuries may be underestimated due to the retrospective design of many studies, some of which do not account for these types of injuries. Avulsion injuries, although less frequent overall, are more commonly reported in studies focusing on specific subpopulations. For example, a study by Warren et al. found a higher incidence of these injuries during ‘after hours’. Additionally, few studies have consistently documented soft tissue injuries associated with dental trauma [27,28,29,30,31].

More than half of the periodontal lesions across both the younger and older age groups were concussions (*p* = 0.004), predominantly located in the frontal area of the dental arch (63.5% and 56.5%, respectively, *p* = 0.019). The literature presents a wide range of reported incidences of oral soft tissue injuries, varying from as low as 3.5% to nearly half of all cases of dental trauma [26,27]. Our findings align with the trend that soft tissue damage is less likely when fewer teeth are involved. Similarly, a study by Škaričić et al. found that soft tissue injuries were more commonly associated with periodontal tissue damage than with injuries to the hard dental structures and pulp [30,31,32,33]. 

In this research study, dental and periodontal traumas were predominantly caused by falls in female subjects (66.7%), while male subjects most frequently experienced trauma from sports activities (49.3%). Specifically, female subjects exhibited a higher incidence of enamel fractures (23.8%), enamel-dentin fractures without pulp exposure (31%), concussions (59.5%), and subluxations (28.6%), and these injuries predominantly occurred in the frontal area. Conversely, male subjects presented with more cases of enamel fractures (23.9%) and enamel-dentin fractures with pulp exposure (22.4%), concussions (58.2%), and avulsions (10.4%), which also mainly occurred in the frontal area (62.7%). Previous research has shown a higher incidence of dental trauma in males compared to females, often attributed to increased sports activity and competition among boys, as well as their generally slower maturity rates. For example, a study by Hang et al. [24] reported tooth trauma in 12% of males versus 8% of females. However, in our study, the difference in the incidence of injuries between males and females was not statistically significant. 

This finding aligns with other studies that have also found no significant gender correlation with dental trauma incidence. Traebert et al. suggest that modern females in Western societies may be exposed to the same risk factors for dental trauma as males, indicating a shift in traditional gender-related risk profiles [4,14,34,35,36,37]. Also, severe dentofacial injuries caused by the use of electric scooters in the UK could be prevented by implementing safety equipment and appropriate legislation [38]. Dental injuries also occur in children with attention deficit hyperactivity disorder (ADHD), who have a higher incidence compared to those without ADHD through a systematic review and meta-analysis of the existing literature [39].

These findings have significant implications for clinical practice, public health initiatives, and future research directions. The identification of age-specific, gender-specific, and residence-related risk factors can inform the development of targeted prevention strategies and educational programs. For example, schools and sports organizations in urban areas might benefit from enhanced dental trauma prevention measures, given the higher risk of radicular fractures among urban residents.

## 5. Conclusions

In conclusion, this study offers valuable insights into the complex interplay of factors influencing dental and periodontal trauma in adolescents and young adults. These findings underscore the importance of age-appropriate, gender-specific, and location-based prevention strategies. Future research must concentrate on longitudinal designs that thoroughly assess the long-term consequences of periodontal and oral injuries and the efficacy of customized therapies based on individual risk factors including gender, age, and physical activity level. The best way to develop and implement prevention initiatives at the local, state, and federal levels is to increase awareness of the prevalence of these traumas among vulnerable populations.

## Figures and Tables

**Table 1:** Socio-demographic characteristics of the group study.

Socio-Demographic Characteristics of the Group Study	N (%)
Age	
10–14 years old	7 (6.42)
14–17 years old	56 (51.37)
18–25 years old	46 (42.2)
Gender	
Female	42 (38.5)
Male	67 (61.5)
Residence	
Urban	59 (54.1)
Rural	50 (45.9)

**Table 2:** Distribution of cases according to etiology, pattern of dental trauma, pattern of periodontal trauma, trauma location, and type of treatment vs. age, gender, and residence.

	Age		Gender		Residence	
	10–17 years	18–25 years	Female	Male	Urban	Rural
**Etiology**						
Domestic accident	15.9%	19.6%	23.8%	13.4%	13.6%	22.0%
Car Accident	19.0%	4.3%	9.5%	14.9%	15.3%	10.0%
Fall	36.5%	43.5%	66.7%	22.4%	42.4%	36.0%
Sports activity	28.6%	32.6%		49.3%	28.8%	32.0%
*p*	0.161		0.000		0.561	
**The pattern of dental trauma**						
Enamel fracture	17.5%	32.6%	23.8%	23.9%	18.6%	30.0%
Enamel-dentin fracture without pulp exposure	23.8%	15.2%	31.0%	13.4%	15.3%	26.0%
Enamel-dentin fracture with pulp exposure	20.6%	10.9%	7.1%	22.4%	13.6%	20.0%
Crown-root fracture	7.9%	30.4%	21.4%	14.9%	22.0%	12.0%
Root fracture	20.6%	8.7%	14.3%	16.4%	22.0%	8.0%
Alveolar fracture	9.5%	2.2%	2.4%	9.0%	8.5%	4.0%
*p*	0.004		0.075		0.090	
**The pattern of periodontal trauma**						
Concussion	54.0%	65.2%	59.5%	58.2%	57.6%	60.0%
Subluxation	17.5%	26.1%	28.6%	16.4%	13.6%	30.0%
Luxation	9.5%	4.3%	4.8%	9.0%	10.2%	4.0%
Extrusion	7.9%	2.2%	4.8%	6.0%	8.5%	2.0%
Avulsion	11.1%	2.2%	2.4%	10.4%	10.2%	4.0%
*p*	0.137		0.309		0.079	
**Trauma location**						
Frontal zone	63.5%	56.5%	57.1%	62.7%	57.6%	64.0%
Lateral zone	25.4%	43.5%	38.1%	29.9%	33.9%	32.0%
Combined	38.1%	13.0%	19.0%	32.8%	23.7%	32.0%
*p*	0.019		0.620		0.590	
**Type of treatment**						
Conservative	81.0%	93.5%	90.5%	83.6%	79.7%	94.0%
Surgical	19.0%	6.5%	9.5%	16.4%	20.3%	6.0%
*p*	0.061		0.309		0.030	

**Table 3:** Multivariate regression analysis of the predisposition to dental trauma according to age, gender, and residence.

		B	Std. Error	Sig.	OR	95% Confidence Interval for Exp (B)	
						Lower Bound	Upper Bound
Pattern of trauma							
Enamel-dentin fracture without pulp exposure	Intercept	1.609	1.349	0.233			
	Age	−1.235	0.628	0.049	0.291	0.085	0.997
	Gender	−1.045	0.623	0.094	0.352	0.104	1.193
	Residence	0.225	0.615	0.715	1.252	0.375	4.181
Enamel-dentin fracture with pulp exposure	Intercept	−1.385	1.669	0.407			
	Age	−1.142	0.669	0.088	0.319	0.086	1.186
	Gender	0.987	0.770	0.200	2.683	0.594	12.125
	Residence	−0.136	0.643	0.833	0.873	0.248	3.080
Crown-root fracture	Intercept	0.969	1.406	0.491			
	Age	0.739	0.674	0.273	2.094	0.559	7.840
	Gender	−0.124	0.638	0.846	0.884	0.253	3.087
	Residence	−1.091	0.644	0.090	0.336	0.095	1.187
Root fracture	Intercept	2.022	1.494	0.176			
	Age	−1.425	0.719	0.047	0.241	0.059	0.984
	Gender	0.089	0.694	0.898	1.093	0.280	4.261
	Residence	−1.449	0.716	0.043	0.235	0.058	0.955
Alveolar fracture	Intercept	−0.983	2.484	0.692			
	Age	−1.901	1.167	0.103	0.149	0.015	1.472
	Gender	1.206	1.187	0.309	3.341	0.326	34.186
	Residence	−1.263	0.955	0.186	0.283	0.044	1.840

## Data Availability

The original contributions presented in the study are included in the article, further inquiries can be directed to the corresponding author/s.
